# Effects of stenosis and aneurysm on blood flow in stenotic-aneurysmal artery

**DOI:** 10.1016/j.heliyon.2023.e17788

**Published:** 2023-06-29

**Authors:** Azad Hussain, Muhammad Naveel Riaz Dar, Elsayed M. Tag-eldin

**Affiliations:** a Department of Mathematics, University of Gujrat, Gujrat 50700,Pakistan; b Faculty of Engineering and Technology, Future University in Egypt, New Cairo, 11835, Egypt

**Keywords:** Blood flow, Stenotic artery, Newtonian fluid, Finite difference discretization, Aneurysm, Unsteady flow

## Abstract

Blood is indeed a suspension of the different type of cells along with shear thinning, yield stress and viscoelastic characteristics, which can be expressed by Newtonian and a lot of non-Newtonian models. Choosing Newtonian fluid as a sample, an unsteady solver for Newtonian fluid is constructed to determine the transient flow of blood in the obscure region. In this probe, the computational unsteady flow of blood in artery with aneurysm and symmetric stenosis has been considered, which is novelty of current research. The results of this investigation can be applied to detect stenotic-aneurysmal diseases and enhance knowledge of the stenotic-aneurysmal artery, which may increase the understanding of medical science. The blood artery is modeled as a circular tube having a 0.3-m radius and a 2-m length along the horizontal axis. The velocity of blood is taken at 0.12 ms^−1^ so that the geometry satisfies the characteristics of the blood vessel. The governing mass and momentum equations are then solved by finite difference technique of discretization. In this research, important variations in blood pressure and velocity at stenosis and aneurysms in the artery are found. The significant influences on blood flow of the stenotic-aneurysmal artery for pressure and velocity profiles of blood are displayed graphically for the Newtonian model.

## Abbreviations

p: Pressure of Fluid (kgm^−1^s^−2^)
r: Radial direction (m)
t: Time (sec)
u: Velocity of fluid (m/s)
$u_{1}$: Radial velocity component (m/s)
$u_{2}$: Tangential velocity component (ms^−1^)
μ: Fluid Reference viscosity (kg $m^{-1}s^{-1})$
X: Axial direction
ρ: Fluid Density
$U_{0}$: Normal inflow velocity (m $s^{-1})$
θ: Tangential direction (rad)

## 1 Introduction

In view of the current record of the WHO (World Health Organization), 2017 [1], infections related to the cardiovascular system are the cause of the maximum number of problems, misery, and fatalities over the world. Nearly 31% of the total casualties are documented because of these cardiovascular diseases. Cardiovascular diseases include coronary heart malady, rheumatic heart malady, cerebrovascular malady, congenital heart malady, and peripheral heart malady, along with another diseases. The main cause for aforesaid diseases is the changing in the position of the small-density lipoproteins and the cholesterol inner side of the lumen, that cause in the hardness of arteries, that is known as atherosclerosis. The aforesaid malady grows with time and constricts the area in the artery, declaring a hemodynamic narrowing, labelled a stenosis [2], that minimizes the transportation of blood to various body organs. Human blood is a multi-phase mixture of cells (platelets, leukocytes, and erythrocytes) in plasma. All the researchers agreed that plasma is a viscous fluid [3]; Anyhow, blood as a whole shows non-Newtonian behavior attributable to the mixtures present which substantially modify its rheological characteristics [4]. The transportation of blood by large arteries can be speculated as a Newtonian fluid. However, in short capillaries or arteries, blood shows a very strong non-Newtonian behavior. Atebek and Ling [5] observed that the other essential function of blood flow in arteries is pulsatile in nature. Lubna Sarwar et al. [6] mathematically investigated the blood flow by stenosed channel. Khaled S. Mekheimer et al. [7,8] explored the tapered artery with nanofluid. S. Nadeem et al. [9] examined the nano-fluid flow in a catheterized stenotic artery along with the porous wall. Saeed Ehsan Awan et al. [[10], [11], [12], [13]] Numerically investigate the micropolar nanofluid between two plates along with MHD and electric hall effect, peristaltic flow with internal heat generation, nanofluid flow with nonlinear radiative heat transfer and solar energy, and magnetic impact on ciliary induced hybrid nanoparticles. K. Ganesh Kumar et al. [14] examine the effect of various hybrid nano fluid. Mohammad Rahimi-Gorji et al. [15] investigated the peristaltic flow along with nano fluid.

A huge number of articles have been accomplished to explain the hemodynamics in capillaries or arteries, assuming blood is either a non-Newtonian or Newtonian fluid. The flow through a stenotically affected artery is studied by Ponalagusamy [16]. It has diverted the attention of a number of researchers, and they further interpreted it mathematically to help other researchers in biology and medicine. The stenosis may be formed in the blood vessel that transporting blood to the brain, which is the carotid artery, or it may be in the artery that transports blood to the kidneys, known as the renal artery. Chaturani et al. [17] constructed the non-Newtonian type of blood flow models. They analyze the stenosis effect on blood flow inside the artery. Different mathematical fluid models that include non-Newtonian characteristics are developed to explore the stenosis vessel study. The various models are presented in Refs. [[18], [19], [20]]. Mandal and Chakravarty [21] calculated the time dependent blood flow in an overlapping stenotic artery by taking into account blood as a non-Newtonian fluid and using the finite difference method (FDM). N. S. Sweed et al. [22] investigated the change in bloodstream along with mass and heat transfer by asymmetric stenotic artery. Mandal and Chakravarty [23] developed the mathematical understanding for blood flow in the artery, assuming it as a Newtonian fluid adopting the clinically sensible the time variant configuration of the overlapping stenosis display in the lumen of the artery and along with the vascular wall flexible deformability. El Kot and Mekheimer [24] examined the transfer of mass, heat, and momentum in shear thinning Sisko blood flow in the stenosis affected artery along with the effects of cross diffusion. Lubna S. et al. [25] explored the effects of nanoparticles in stenotic arteries. Khaled S. Mekheimer et al. [26] examined the elasticity of stenosis to check the contribution of non-Newtonian flow by arterial tube. Arindam Bit et al. [27] explored the 3-dimensional analysis of stenosed artery. Ali, R., Asjad, M.I., Aldalbahi, A. et al. [28] studied the flow of Maxwell nano fluid with pressure gradient.

An aneurysm is expansion in the vessel, including an increment in diameter 50% above in comparison with the normal blood vessel diameter. It is due to non-regular growth or very weak walls of blood vessels, which have a balloon or bubbled format. Cerebral aneurysms, peripheral aneurysms, and aortic aneurysms are the most common in the human body. The nature and main cause of aneurysms are still an important issue of research, and nearly 75% of aneurysm patients die before touching the hospital. The investigation of blood flow has captivated a lot of medical researchers, bioengineers, etc. In the past few years, because of its clear effect on many human cardiovascular maladies like bleeding, arteriosclerosis, high blood pressure, stroke and kidney damage. A number of researchers [29,30] have analyzed aneurysms from different perspectives and still it has space to be investigate in this sector. Thurston [31] studied the relationship among the viscoelasticity and the shear rate of blood. Wille [32] further analyzed the existence of the vortex and the pulsatile blood flow in aneurysm. Mandal, M.S. et al. [33] studied the cross-viscosity model in aneurysmal artery for pulsatile flow.

Hemodynamics in ill arteries is even further complicated by the interaction and pairing of aneurysm and stenosis. By using the Bingham model to represent blood flow, Pincombe et al. [29] looked into the impact of blood vessel stenosis and dilatations in different combination on the reactance impedance to flow. A. M. Abdelwahab et al. [34] explored the numerical results of electro-osmotic type forces on micropolar bloodstream by stenotic and aneurysmal carotid. According to Prasad et al. [35], Jeffery fluid can flow steadily across a tube that has both compression and dilatations. To assess the flow pattern, multiple experimental and theoretical research of fluid dynamics utilising numerous geometric shapes of narrowing or expansion are highlighted in Refs. [[36], [37], [38]]. Khaled S. Mekheimer et al. [39] studied the double diffusion and electroosmotic impact on flow of nano-blood flow by aneurysmal and stenotic affected artery. The study of unsteady flow of blood in stenotic and aneurysmal artery is not analyzed in the literature. So, this is the novelty of the current research. Mukhopadhyay, S. et al. [40] examined the pulsatile blood flow with shear depending viscosity in stenosed artery.

In this portray, a mathematical review of blood flow along with the stenotic and aneurysmal vessel has been analyzed to investigate the influence of narrowing of the blood vessel, expansion of the blood vessel and the intensity of aneurysm for the Newtonian case. A lot of Numerical techniques used to solve such problems are examined [[41], [42], [43]]. The detailed scientific inspection is carried out. The 3-dimensional model geometry is taken to investigate the unsteady flow of blood. Which is the novelty of the considered problem. The mathematical equations for the flow of blood are considered for unsteady 2D cylindrical flow. The governing mass and momentum equations are solved finite difference numerical technique. The effect of stenosis and aneurysm on flow blood velocity and pressure is shown in graphs. The results may be used in medical for understanding the blood flow in such narrowing arteries.

The paper's remaining content is organized in the following manner: The system mathematical formulation is presented in Section 2, the procedure of the numerical solution is given in Section 3, the outcomes of developed scheme and result discussions are explained in Section 4, while conclusion explained in Section 5.

## 2 Mathematical formulation

**Fig. 1:**
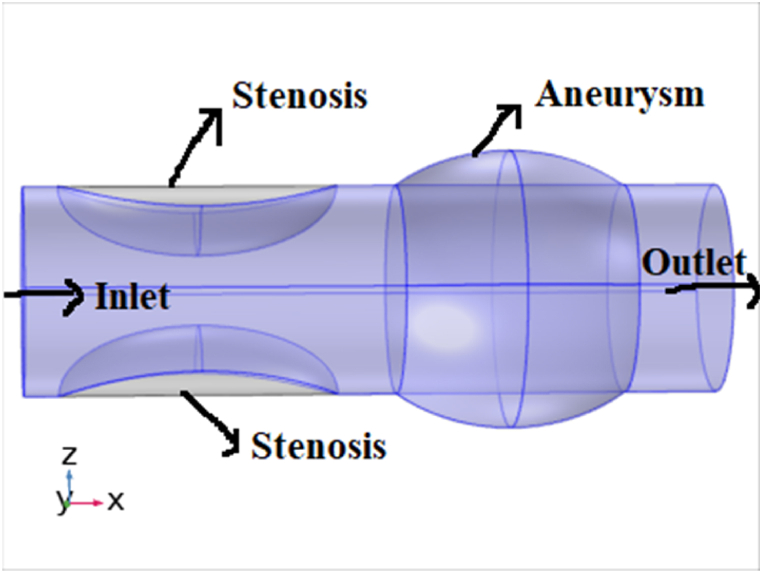
Geometry of the flow through the stenotic-aneurysmal artery.

A swelled and stenotic affected artery is modeled. The unsteady two-dimensional incompressible Newtonian flow is considered. The considered flow model in the current investigation is shown in Fig. 1. We supposed that the blood is consistently implant or extracted with constant speed $U_{0}$ from the aneurysmatic and stenotic affected artery.

The equation that portrays the geometry of the wall is written as in Equ. (1)

(1) $$\bar{R}\left(x\right)=\left\{ \begin{array}{c} 1-\frac{\delta^{*}}{2R_{0}}\left(1+\cos\left[\frac{2\pi }{l_{i}}\left(\bar{x}-\beta_{i}-\frac{l_{i}}{2}\right)\right]\right),\beta_{i}\leq \bar{x}\leq \beta_{i}+l_{i},i=1,2.\\ 1,otherwise. \end{array}\right.$$

According to a previous expression, $l_{i}$ is the irregular section length, $R_{0}$ is the regular artery radius, and $\beta_{i}$ is the distance of the irregular portion from the origin. $\delta^{*}$ is abnormal segment's critical height, that emerges at two specific locations $\bar{x}=\beta_{1}+\frac{l_{0}}{2}$ and $\bar{x}=\beta_{2}+\frac{l_{0}}{2}$.

The compact form of governing equations for the blood flow by the aneurysmal and stenotic affected artery is mathematically identified as Eqs. (2), (3):

(2) ρ∇.(u)=0,(Incompressibleflow)

(3) $$\rho \frac{\partial u}{\partial t}+\rho \left(u.\nabla \right)u=\mathrm{div}\left[-pI]+\mathrm{div}[K\right]+F\text{.}$$

where $K={\mu (grad\left(u\right)+\left(grad\left(u\right)\right)}^{T})$.

The aforesaid compact equations for the velocity vector field $u=\left(u_{1}\left(r,\theta ,x,t\right),u_{2}\left(r,\theta ,x,t\right),u_{3}\left(r,\theta ,x,t\right)\right)$, by ignoring the body forces are interpreted as:

(4) $$\frac{\partial u_{1}}{\partial r}+\frac{u_{1}}{r}+\frac{1}{r}\frac{\partial u_{2}}{\partial \theta }+\frac{\partial u_{3}}{\partial x}=0\text{,}$$

(5) $$\rho \left(\frac{\partial u_{1}}{\partial t}+u_{1}\frac{\partial u_{1}}{\partial r}+\frac{u_{2}}{r}\frac{\partial u_{1}}{\partial \theta }-\frac{{u_{2}}^{2}}{r}+u_{3}\frac{\partial u_{1}}{\partial x}\right)=-\frac{\partial p}{\partial r}+\mu \left(\frac{1}{r}\frac{\partial \left(rK_{rr}\right)}{\partial r}+\frac{1}{r}\frac{\partial \left(K_{r\theta }\right)}{\partial \theta }-\frac{K_{\theta \theta }}{r}+\frac{\partial \left(K_{rx}\right)}{\partial x}\right)\text{,}$$

(6) $$\rho \left(\frac{\partial u_{2}}{\partial t}+u_{1}\frac{\partial u_{2}}{\partial r}+\frac{u_{2}}{r}\frac{\partial u_{2}}{\partial \theta }-\frac{u_{1}u_{2}}{r}+u_{3}\frac{\partial u_{2}}{\partial x}\right)=-\frac{1}{r}\frac{\partial p}{\partial \theta }+\mu \left(\frac{1}{r^{2}}\frac{\partial \left(r^{2}K_{\theta r}\right)}{\partial r}+\frac{1}{r}\frac{\partial \left(K_{\theta \theta }\right)}{\partial \theta }+\frac{\partial \left(K_{\theta x}\right)}{\partial x}\right)\text{,}$$

(7) $$\rho \left(\frac{\partial u_{3}}{\partial t}+u_{1}\frac{\partial u_{3}}{\partial r}+\frac{u_{2}}{r}\frac{\partial u_{3}}{\partial \theta }+u_{3}\frac{\partial u_{3}}{\partial x}\right)=-\frac{\partial p}{\partial x}+\mu \left(\frac{1}{r}\frac{\partial \left(rK_{rx}\right)}{\partial r}+\frac{1}{r}\frac{\partial \left(K_{\theta x}\right)}{\partial \theta }+\frac{\partial \left(K_{xx}\right)}{\partial x}\right)\text{.}$$

Where $K_{rr}$, $K_{r\theta }\text{,}$ $K_{rx}\text{,}$ etc. are the components of stress tensor. By using the values of all the components, equations (4), (5), (6) are reduced as:

(8) $$\rho \left(\frac{\partial u_{1}}{\partial t}+u_{1}\frac{\partial u_{1}}{\partial r}+\frac{u_{2}}{r}\frac{\partial u_{1}}{\partial \theta }-\frac{{u_{2}}^{2}}{r}+u_{3}\frac{\partial u_{1}}{\partial x}\right)=-\frac{\partial p}{\partial r}+\mu \left(\frac{2}{r}\frac{\partial u_{1}}{\partial r}+2\frac{\partial^{2}u_{1}}{{\partial r}^{2}}+\frac{1}{r}\frac{\partial^{2}u_{2}}{\partial \theta \partial r}+\frac{1}{r^{2}}\frac{\partial^{2}u_{1}}{{\partial \theta }^{2}}-\frac{2}{r^{2}}\frac{\partial u_{2}}{\partial r}-\frac{2u_{1}}{r^{2}}+\frac{\partial^{2}u_{3}}{\partial x\partial r}+\frac{\partial^{2}u_{1}}{{\partial x}^{2}}\right)\text{,}$$

$$\rho \left(\frac{\partial u_{2}}{\partial t}+u_{1}\frac{\partial u_{2}}{\partial r}+\frac{u_{2}}{r}\frac{\partial u_{2}}{\partial \theta }-\frac{u_{1}u_{2}}{r}+u_{3}\frac{\partial u_{2}}{\partial x}\right)=-\frac{1}{r}\frac{\partial p}{\partial \theta }+\mu (\frac{2}{r}\frac{\partial u_{2}}{\partial r}+\frac{\partial^{2}u_{2}}{{\partial r}^{2}}+\frac{1}{r^{2}}\frac{\partial u_{2}}{\partial \theta }+\frac{1}{r}\frac{\partial^{2}u_{1}}{\partial r\partial \theta }+\frac{2}{r^{2}}\frac{\partial^{2}u_{2}}{{\partial \theta }^{2}}$$

(9) $$+\frac{2}{r^{2}}\frac{\partial u_{1}}{\partial \theta }+\frac{1}{r}\frac{\partial^{2}u_{3}}{\partial x\partial \theta }+\frac{\partial^{2}u_{2}}{{\partial x}^{2}})\text{,}$$

(10) $$\rho \left(\frac{\partial u_{3}}{\partial t}+u_{1}\frac{\partial u_{3}}{\partial r}+\frac{u_{2}}{r}\frac{\partial u_{3}}{\partial \theta }+u_{3}\frac{\partial u_{3}}{\partial x}\right)=-\frac{\partial p}{\partial x}+\mu \left(\frac{1}{r}\frac{\partial u_{3}}{\partial r}+\frac{\partial^{2}u_{3}}{{\partial r}^{2}}+\frac{1}{r}\frac{\partial u_{1}}{\partial x}+\frac{\partial^{2}u_{1}}{{\partial x}^{2}}+\frac{1}{r^{2}}\frac{\partial^{2}u_{3}}{{\partial \theta }^{2}}+\frac{1}{r}\frac{\partial^{2}u_{2}}{\partial \theta \partial x}+2\frac{\partial^{2}u_{3}}{{\partial x}^{2}}\right)\text{.}$$

In present study, the velocity component $u_{2}=0\text{,}$ and flow is not depending on the angle θ, so, equations (4), (8), (8), (9), (10) reduce to:

(11) $$\frac{\partial u_{1}}{\partial r}+\frac{u_{1}}{r}+\frac{\partial u_{3}}{\partial x}=0\text{,}$$

(12) $$\rho \left(\frac{\partial u_{1}}{\partial t}+u_{1}\frac{\partial u_{1}}{\partial r}+u_{3}\frac{\partial u_{1}}{\partial x}\right)=-\frac{\partial p}{\partial r}+\mu \left(\frac{2}{r}\frac{\partial u_{1}}{\partial r}+2\frac{\partial^{2}u_{1}}{{\partial r}^{2}}-\frac{2u_{1}}{r^{2}}+\frac{\partial^{2}u_{3}}{\partial x\partial r}+\frac{\partial^{2}u_{1}}{{\partial x}^{2}}\right)\text{,}$$

(13) $$0=\frac{\partial p}{\partial \theta }\text{,}$$

(14) $$\rho \left(\frac{\partial u_{3}}{\partial t}+u_{1}\frac{\partial u_{3}}{\partial r}+u_{3}\frac{\partial u_{3}}{\partial x}\right)=-\frac{\partial p}{\partial x}+\mu \left(\frac{1}{r}\frac{\partial u_{3}}{\partial r}+\frac{\partial^{2}u_{3}}{{\partial r}^{2}}+\frac{1}{r}\frac{\partial u_{1}}{\partial x}+\frac{\partial^{2}u_{1}}{{\partial x}^{2}}+2\frac{\partial^{2}u_{3}}{{\partial x}^{2}}\right)\text{.}$$

equations (11), (12), (13), (14) are solved computationally by finite element discretization technique.

The initial and conditions for the boundary are defined as below in equation (15):

(15) $$u_{1}=u_{2}=u_{3}=0\;at\;t=0\text{,}$$

Inlet:

$u\left(t,r,x\right)=-U_{0}n$, where $U_{0}$ is the normal inflow velocity whose average value inside the normal artery is $0.12ms^{-1}\text{.}$ The minus sign describes the flow is from higher concentration to lower concentration.

Outlet:

(16) $$\left[-pI+K\right]n=-\hat{p_{0}}n,where\;\hat{p_{0}}\leq p_{0}\text{.}$$

The boundary condition for the suppress backflow at the outlet is $p_{0}=16000pa$ for the normal blood pressure in the human body as in equation (16).

Wall:

u=0, no slip on the wall, i.e. velocity is zero on the wall.

The values of the pertaining parameters used in the calculation, the density of blood in the artery is $1060\frac{kg}{m^{3}}$, the dynamic viscosity of blood is 0.003pa.s, the thermal conductivity of blood is $0.52wm^{-1}k^{-1}$ and the heat capacity of blood by considering constant pressure is $3617J{kg}^{-1}k^{-1}$.

## 3 Procedure of the numerical solution

**Fig. 2:**
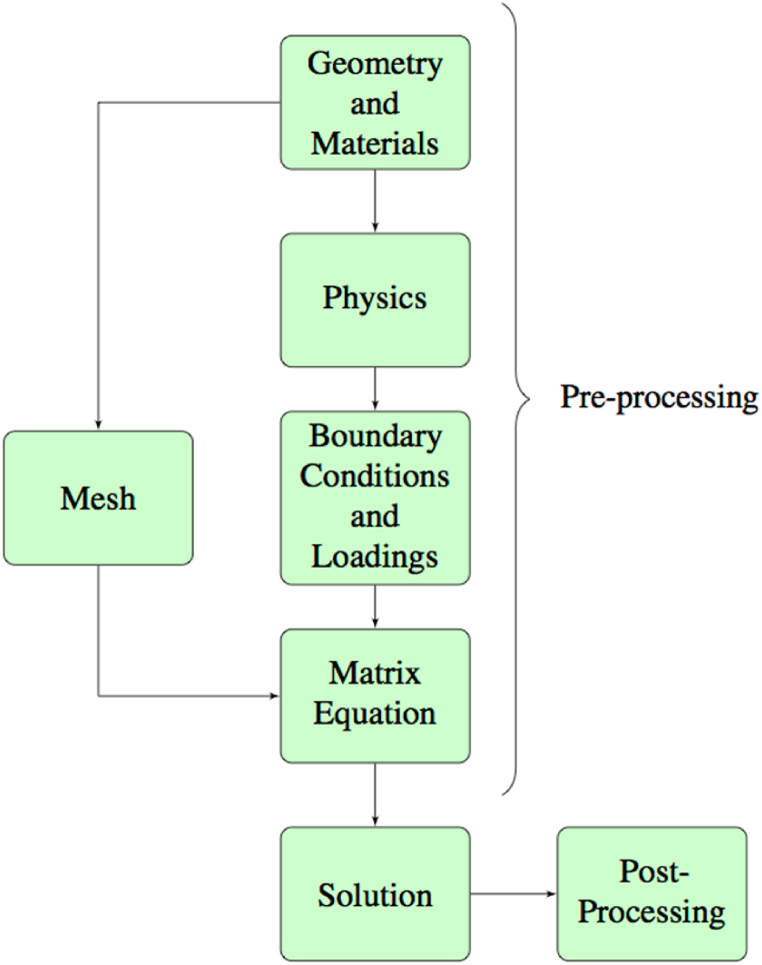
Basic Procedure to conduct finite element analysis.

As shown in Fig. 2, the finite element analysis is carried out by following a set of steps.

The procedure can be broken down into three simple steps: pre-processing, solver, and post-processing [38]. Building the model is made possible by the pre-processing stage. It is necessary to break it down into smaller sub-steps because it includes all of the information about the FEM application for the interconnect study. The creation of the interconnect geometry (1D, 2D, or 3D) is the first step in the process. It is used to represent the domain under study and give it a set of material properties. Then, by giving the model the underlying physics (or Multiphysics), mathematical equations, and finite element formulation, the physical environments of the question under examination are produced. The latter is typically tucked away in the commercial software's core. The matrix equation that governs the model is then determined by applying the proper loadings, boundary, and initial conditions to the domain under study and discretizing it into finite elements.

**Fig. 3:**
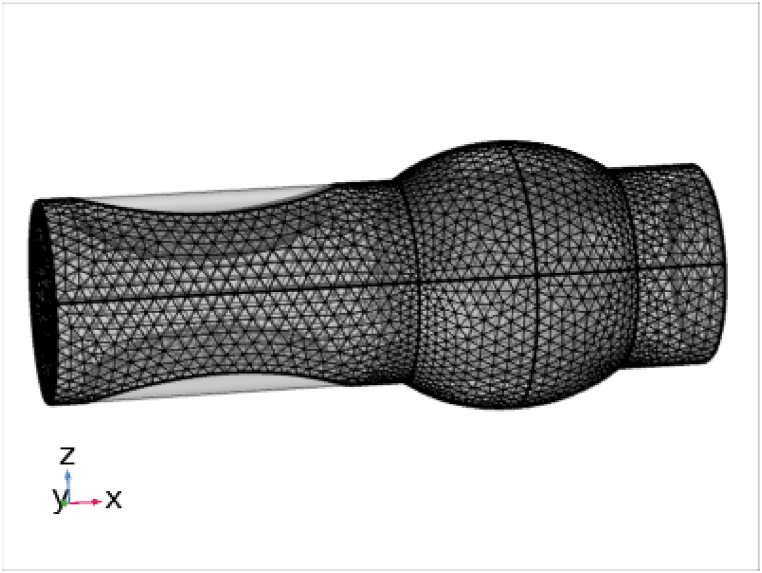
Normal size mesh.

Physics-controlled mesh which is taken as normal mesh is applied to obtain the results. Mesh has great importance in computational fluid dynamics because the quality of mesh determines the accuracy and convergence of the solution. The mesh is automatically generated by using physics control mesh. The normal type of mesh is shown in Fig. 3. This method is used for flow of blood along with nanoparticle in cartesian coordinates as [44,45].

## 4 Discussion and outcomes

The blood flow in the arteries is crucial and demanding. It is really hard to investigate the flow through arteries mathematically. In the system of arteries, the most occurring maladies are narrowing and swelling of the wall of blood vessels which are calling as stenosis and aneurysm respectively. To find the pressure and velocity of blood by the narrow and swelled artery, the mathematical model is studied. The x-axis is used to conduct the analysis. Different locations along the x-axis are chosen for the cut planes. so that it would be possible to comprehend how blood pressure and velocity behave. It has also been noted what the variation in velocity and pressure will be at the same position with changing in time. The results about velocity and pressure will be helpful to control the blood pressure and overcome different diseases.

### 4.1 Velocity profile

**Fig. 4:**
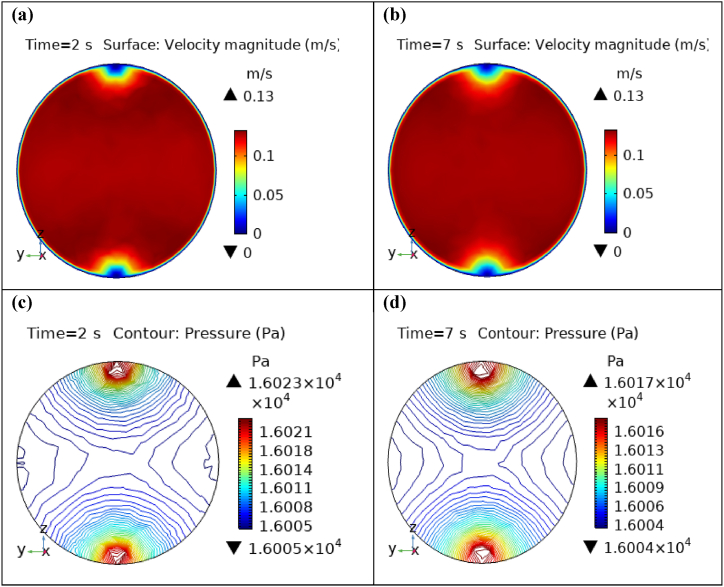
Velocity and Pressure cut planes at x=0.1m for t=2s and t=7s.

**Fig. 5:**
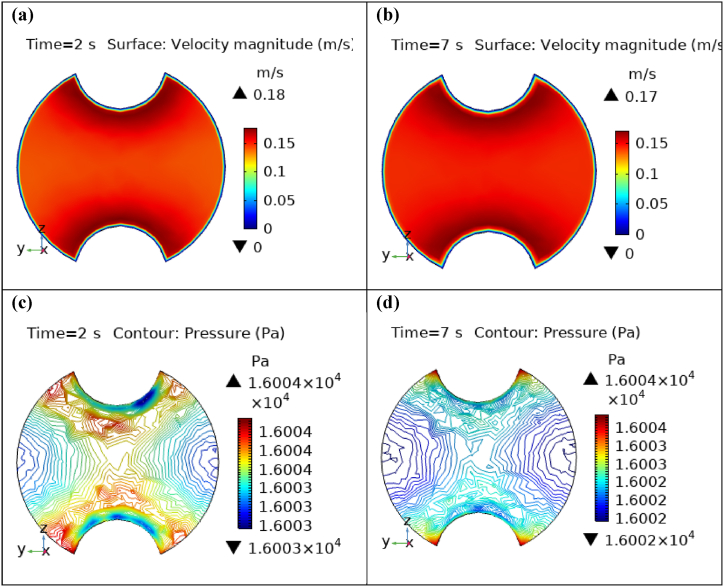
Velocity and Pressure cut planes at x=0.2m for t=2s and t=7s.

**Fig. 6:**
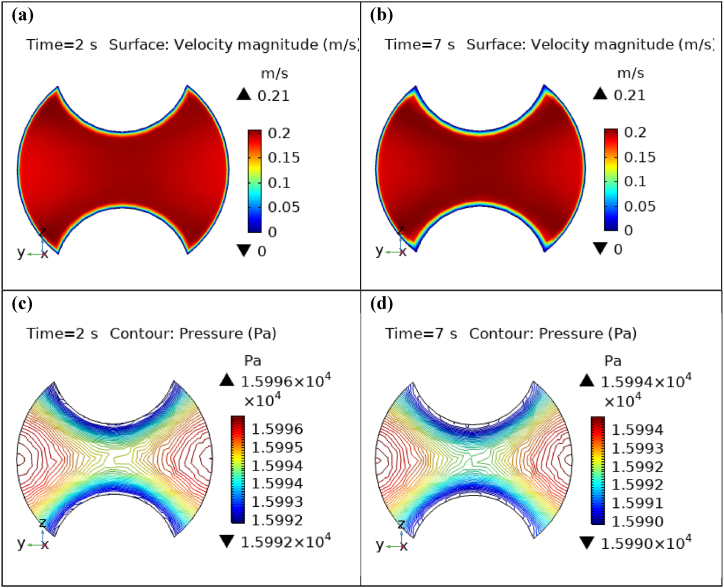
Velocity and Pressure cut planes at x=0.6m for t=2s and t=7s.

**Fig. 7:**
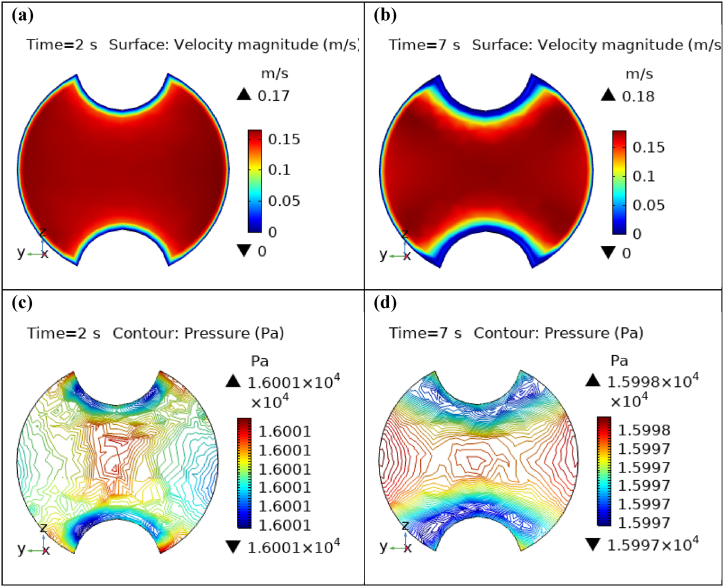
Velocity and Pressure cut planes at x=0.8m for t=2s and t=7s.

**Fig. 8:**
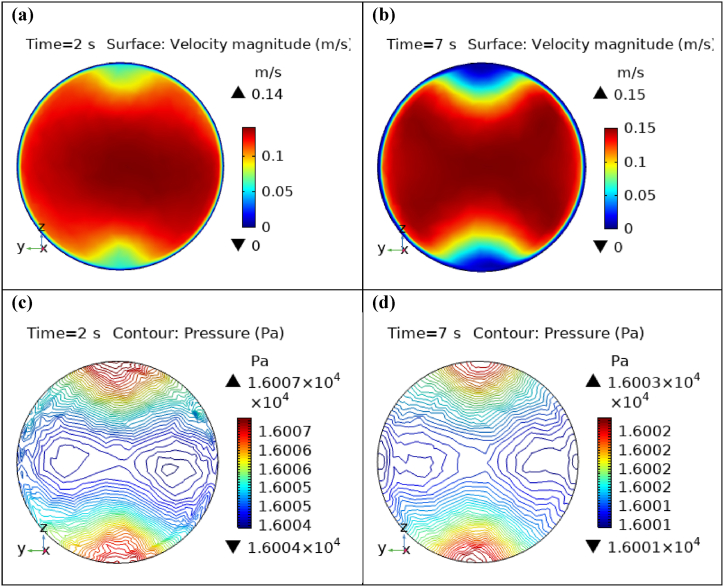
Velocity and Pressure cut planes at x=1m for t=2s and t=7s.

**Fig. 9:**
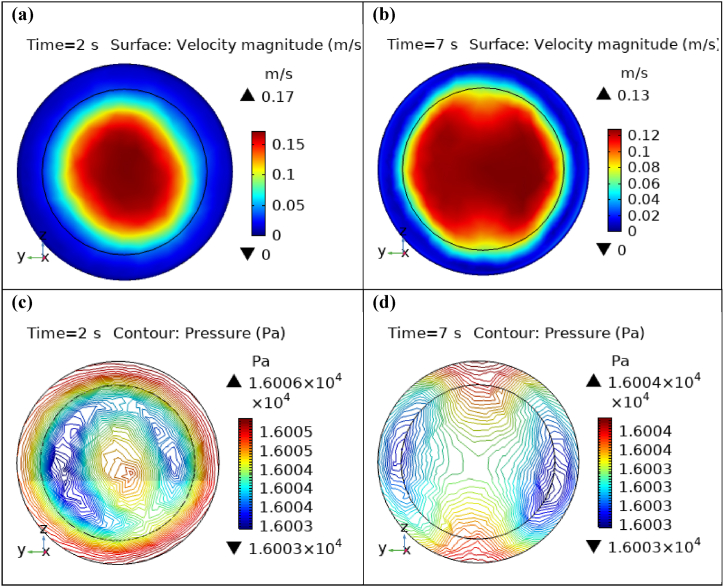
Velocity and Pressure cut planes at x=1.3m for t=2s and t=7s.

**Fig. 10:**
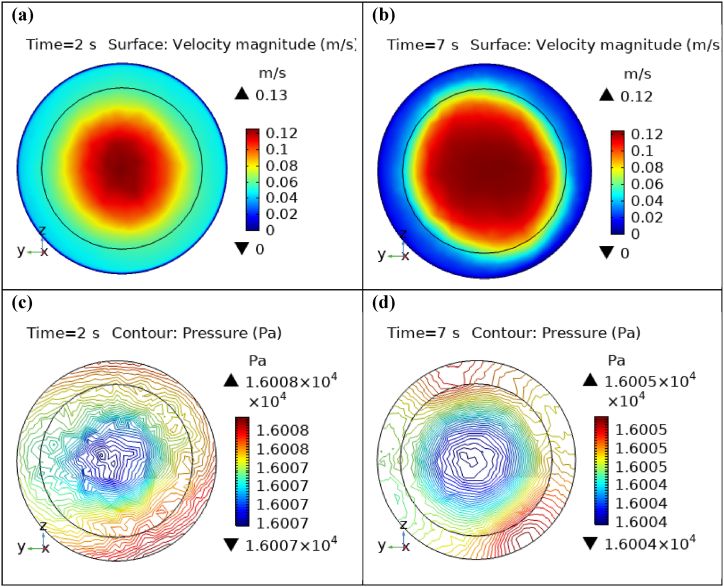
Velocity and Pressure cut planes at x=1.5m for t=2s and t=7s.

**Fig. 11:**
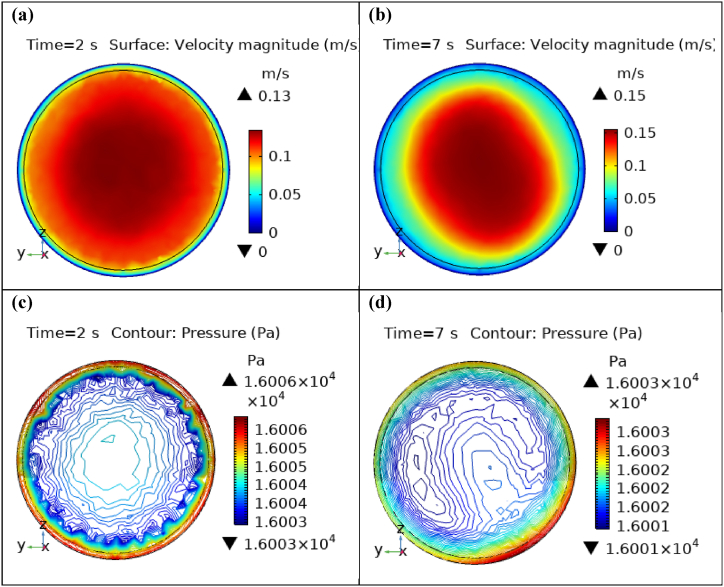
Velocity and Pressure cut planes at x=1.7m for t=2s and t=7s.

**Fig. 12:**
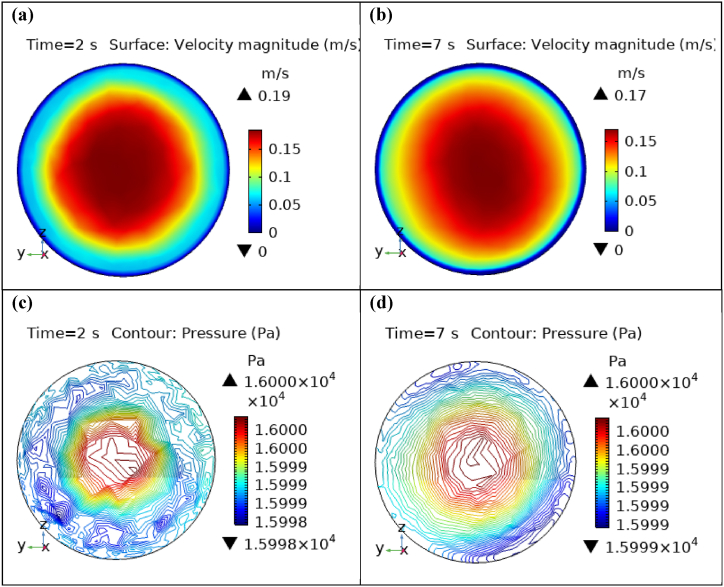
Velocity and Pressure cut planes at x=1.9m for t=2s and t=7s.

**Fig. 13:**
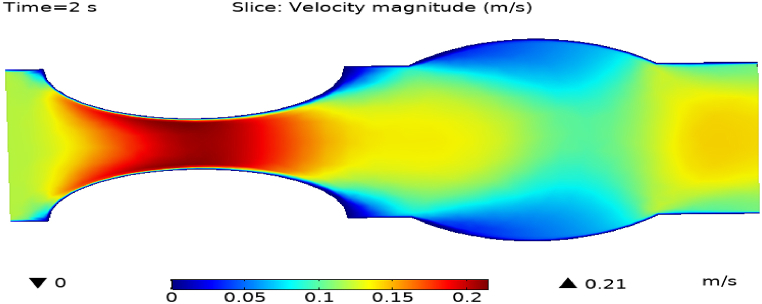
Velocity field for t=2s..

**Fig. 14:**
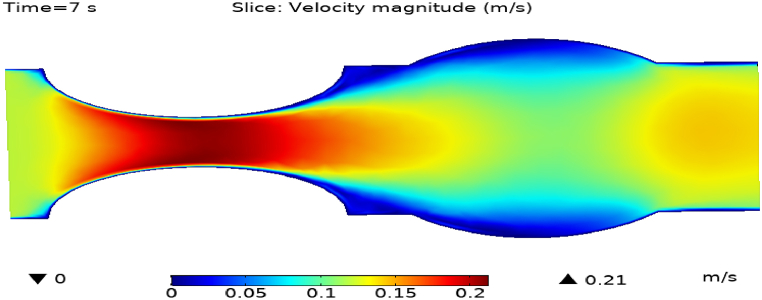
Velocity field for t=7s..

Fig. 4(a) and (b) describe the cut planes for the velocity profile at x=0.1m distance for t=2s and t=7s. Velocity is zero at the point where stenosis is just started and it little bit changes with time. Fig. 5(a) and (b) display the velocity cut planes at x=0.2m distance for t=2s and t=7s. The maximum velocity for t=2s and t=7s is $0.18ms^{-1}$ and $0.17ms^{-1}$ respectively. Velocity is reducing with time. The velocity decreases by decreasing the diameter. Figs. 6(a) and 7(a), 8(a), 9(a), 10(a), 11(a) and 12(a) are the velocity cut planes for t=2s at different distances along the x-axis. That shows the velocity behavior at corresponding positions. Figs. 6(b), 7(b) and 8(b), 9(b), 10(b), 11(b) and 12(b) display the cut planes of velocity field for t=7s at different distances along the x-axis. Fig. 12, Fig. 13, Fig. 14 show how velocity changes throughout the artery with time. The velocity is initially increases from start to maximum height of the stenosis. When the height of stenosis is increasing the velocity decreases and it is continuously decreases to mid-point of the aneurysm. After that it is again increases. Table 1, Table 2 display the numeric results of the variation of maximum velocity along the x-axis.

**Table 1:** Variation in velocity and pressure at t=2s.

Distance (x)	Maximum Velocity (ms^−1^)	Maximum Pressure (Pa)	Distance (x)	Maximum Velocity (ms^−1^)	Maximum Pressure (pa)
0.1 m	0.13	16023	1.3 m	0.17	16006
0.2 m	0.18	16004	1.5 m	0.13	16008
0.6 m	0.21	15996	1.7 m	0.13	16006
0.8 m	0.17	16001	1.9 m	0.19	16000
1 m	0.14	16007

**Table 2:** Variation in velocity and pressure at t=7s.

Distance (x)	Maximum Velocity (ms^−1^)	Maximum Pressure (Pa)	Distance (x)	Maximum Velocity (ms^−1^)	Maximum Pressure (pa)
0.1 m	0.13	16017	1.3 m	0.15	16004
0.2 m	0.17	16004	1.5 m	0.12	16005
0.6 m	0.21	15994	1.7 m	0.15	16003
0.8 m	0.18	15998	1.9 m	0.18	16000
1 m	0.13	16003			

### 4.2 Pressure profile

**Fig. 15:**
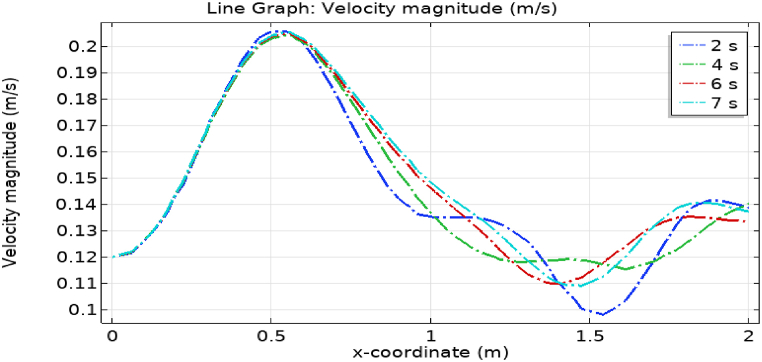
Velocity field line graph.

**Fig. 16:**
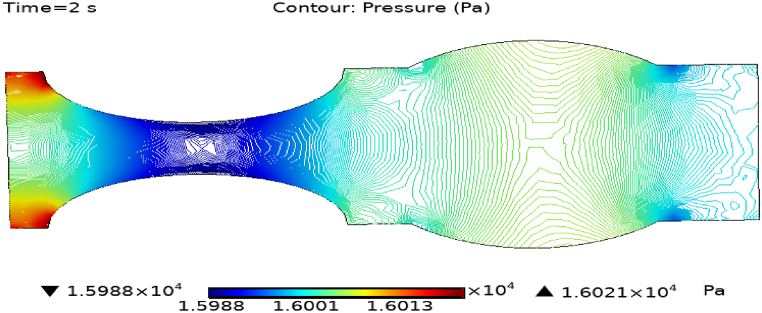
Pressure field for t=2s..

**Fig. 17:**
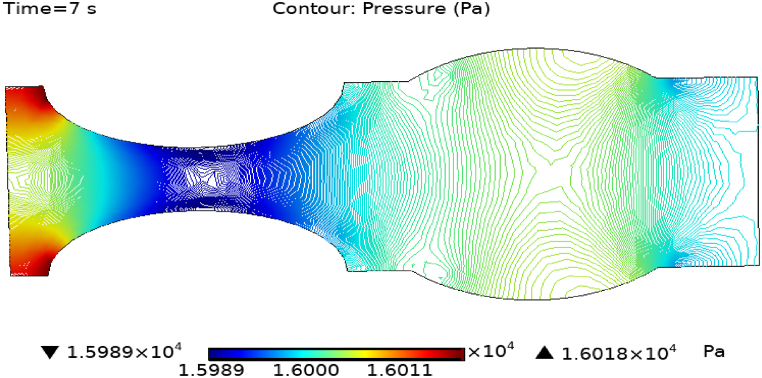
Pressure field for t=7s..

**Fig. 18:**
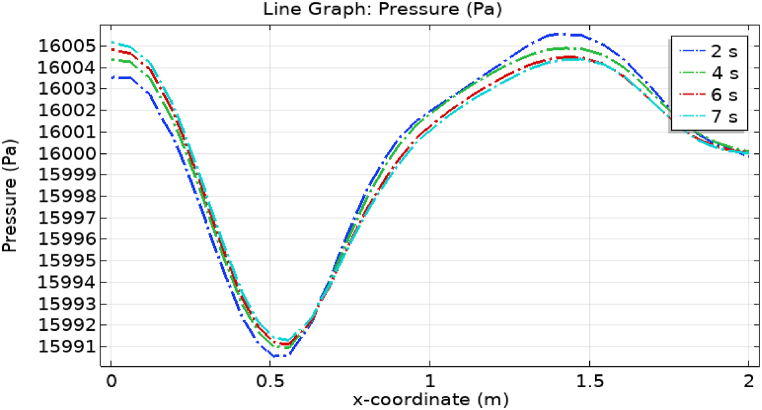
Pressure field line graph.

Fig. 4(c) and (d) shows the cut planes for pressure profiles at x=0.1m distance for t=2s and t=7s. Pressure profile is also changing the little bit with the wall where stenosis is just started. Fig. 5(c) and (d) display the pressure cut planes at x=0.2m distance for t=2s and t=7s. For t=2s pressure shows irregular behavior and for t=3s pressure is the maximum at the edges only. The minimum value of pressure is decreases. The pressure increases by decreasing the diameter. Fig. 6, Fig. 7, Fig. 8, Fig. 9, Fig. 10, Fig. 11, Fig. 12 are the pressure cut planes for t=2s at different distances along the x-axis. That shows the pressure behavior at those specific positions. Fig. 6, Fig. 7, Fig. 8, Fig. 9, Fig. 10, Fig. 11, Fig. 12 display the cut planes of pressure field for t=7s at different distances along the x-axis. Fig. 15, Fig. 16, Fig. 17 display the profile for pressure variation along the x-axis with time. It is clear from the figures that, at the start of stenosis, the pressure is maximum. The pressure is decreasing with increasing the height of stenosis and it is increasing with decreasing the height of stenosis. The pressure is increasing from start of the aneurysm to its maximum height and it decreases with decreasing the height of aneurysm (see Fig. 18). Table 1, Table 2 show the numerical values of the variation of maximum pressure at different distances along the x-axis (see Fig. 18).

## 5 Conclusion

The effects of stenosis and aneurysm on the pulsatile flow velocity and pressure for the artery have been studied numerically and visually. Significant blood flow phenomena are quantitatively analyzed and explained using the CFD technique (Finite Difference Discretization). The most significant characteristics of unsteady blood flow are investigated. An overview of the study's main conclusions is furnished below:

⁃ The velocity of blood increases from the start of stenosis to the mid of stenosis and from the mid to end of the stenosis, the velocity decreases along the axis of symmetry. It decreases with time from the start of stenosis to the mid and increases from the mid to end of stenosis.
⁃ For aneurysm, the velocity increases from the starting point of aneurysm to mid-point of aneurysm and decreases from the mid to end of aneurysm along the axis of symmetry. It is decreasing with respect to time from the start of aneurysm to just before ending the aneurysm, then just before ending it is again start to increase with time.
⁃ The pressure is maximum at the starting point of stenosis and it is decreasing along the axis of symmetry to the mid-point of stenosis. Now from the mid to the end point of stenosis the pressure is increasing. For the region between stenosis and aneurysm the pressure is increasing, and continuously increasing till the mid-point of aneurysm. After that, it is again decreasing.
⁃ The pressure is continuously decreasing for the whole stenotic-aneurysmal artery with respect to time.

In order to comprehend the causes of stenosis and dilatation, as well as to analyze the issue using radiation and magnetohydrodynamic implications, we can examine the physical features in greater detail. This could aid in the treatment of arterial stenosis. We can also study the impact of nanoparticle with this study that helps in the treatment of diseases causes by stenosis and aneurysms.

## Author contribution statement

Azad Hussain: Conceived and designed the experiments; Performed the experiments; Analyzed and interpreted the data; Contributed reagents, materials, analysis tools or data.

Muhammad Naveel Riaz Dar: Conceived and designed the experiments; Performed the experiments; Analyzed and interpreted the data; Contributed reagents, materials, analysis tools or data; Wrote the paper.

Elsayed M. Tag-eldin: Contributed reagents, materials, analysis tools or data.

## Data availability statement

Data will be made available on request.

## Declaration of competing interest

The authors declare that they have no known competing financial interests or personal relationships that could have appeared to influence the work reported in this paper.
